# Surgical excision of peripheral giant cell granuloma of the maxilla: a case report

**DOI:** 10.11604/pamj.2023.44.141.34835

**Published:** 2023-03-20

**Authors:** Narjiss Akerzoul, Babacar Touré

**Affiliations:** 1 Department of Oral Surgery and Oral Medicine, International Faculty of Dental Medicine, College of Health Sciences, International University of Rabat, Rabat, Morocco,; 2 Department of Conservative Dentistry, International Faculty of Dental Medicine, College of Health Sciences, International University of Rabat, Rabat, Morocco

**Keywords:** Peripheral giant cell granuloma, granuloma, maxilla, traumatic extractions, case report

## Abstract

Peripheral giant cell granuloma (PGCG) is described as an elevated lesion that is located mostly on the gingival mucosa and alveolar crest, consecutive to irritative factors and trauma. It predominantly occurs more in the mandible than the maxilla, and it is usually seen in the 4^th^ to the 6^th^ decades. The clinical appearance of this lesion is red-bluish in color, presenting a similar tissue to the one observed in the liver, usually measuring less than 2 cm. The treatment of the PGCG is the surgical excision. The recurrence of this lesion is rarely described in the literature. The present case highlights the importance of considering the traumatic extractions as one of the main uncommon etiologic factors, leading to the development of peripheral giant cell granuloma. It precisely describes the diagnosis, the treatment of a peripheral giant cell granuloma located in maxillary canine-premolar region, occurred consecutively after ancient traumatic extractions of the 13 and 14 since 1 year. This paper also reports a maxillary location of giant cell granuloma, while the literature reports more commonly the mandibular location. This lesion was excised surgically, and healed uneventually, and in which the follow-up didn´t show any sign of recurrence.

## Introduction

Peripheral giant cell granuloma is a non-malignant lesion that develops as a consequence of chronic tissue injury. It has been described as an exophytic lesion characterized by non-malignant nodular swellings that develop as a consequence of chronic tissue injury and an exuberant tissue response originating from periodontal ligament cells [1-4]. Local factors including dental plaque and calculus, food impaction, chronic infections, periodontal disease or periodontal surgery, defective restorations, ill-fitting appliances, and trauma from tooth extractions have been documented as eventual factors incriminated in causing PGCG [4]. It is a relatively unusual lesion that occurs in both young and old patients; however, occurrences seem to occur more frequently in elderly individuals. Only about 20-30% of cases manifest in children aged 1-10, while only 9% of the cases manifest in pediatric populations.

Peripheral giant cell granuloma is often characterized by a painless, smooth surface lesion that can range from a few millimeters to several centimeters. It's typically brown, red, or even purple in hue and may have a sessile or pedunculate implantation base, with a soft to a firm consistency. Microscopically the lesion features an abundance of multinucleated giant cells. This is how it differentiates from other exophytic oral differential diagnoses [3]. The distinction between central and peripheral giant cell granuloma is established through radiographic evaluation - in the former there's the presence of an intrabony benign neoplasm of the jawbone. The purpose of this paper targets surgical treatment of peripheral giant cell granuloma in a 60-year-old female generated by post-traumatic extractions; with no signs of recurrence after two months of follow-up.

## Patient and observation

**Patient information:** a female patient aged 60, reported to the Oral Surgery and Oral Medicine Department, of the Dental Teaching Hospital affiliated to the International Faculty of Dental Medicine of Rabat, complaining of discomfort consecutive to an extraoral inflammation in the superior right maxillary, dating back 1 year. The patient´s medical antecedents showed nothing in particular. The patient was apparently healthy.

**Clinical findings and timeline of the current episode:** the patient reported that the exophytic lesion was basically a small lesion and slowly increased in size over 1 year. According to the patient, the lesion occurred right after having undergone traumatic extractions of residuals 13 and 14. It was accompanied by intermittent pain. There were no other locations of the same type of lesion in any part of the body.

**Figure 1:**
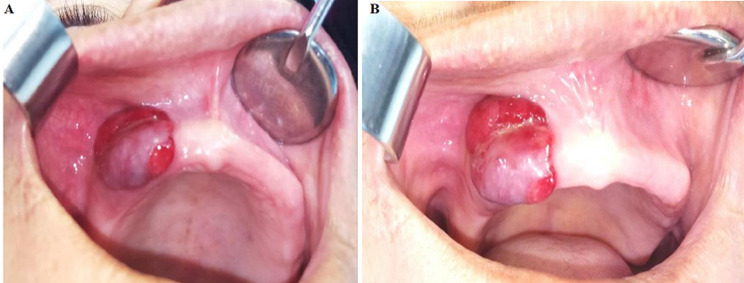
A,B) intraoral examination showing a single blue-reddish swelling on the left maxillary canine-premolar region

**Diagnostic assessment:** the intra-oral examination revealed the presence of a swelling on the left maxillary canine premolars. The lesion measured about 1.5 x 1.5 cm. The exterior of the lesion was lobulated close to 12, 13, and 14. The lesion was firm in consistency and presented a blue-reddish color, with a firm pedicle. The covering mucosa was intact (Figure 1). Panoramic incidence, completed with periapical X-ray showed no bone resorption.

**Diagnosis:** based on clinical and radiological patterns, we primarily concluded a peripheral giant cell granuloma as there was no bone resorption on the imaging which differentiates the peripheral to central giant cell granuloma. Our primary diagnosis was then subject to confirmation after histological examination.

**Figure 2:**
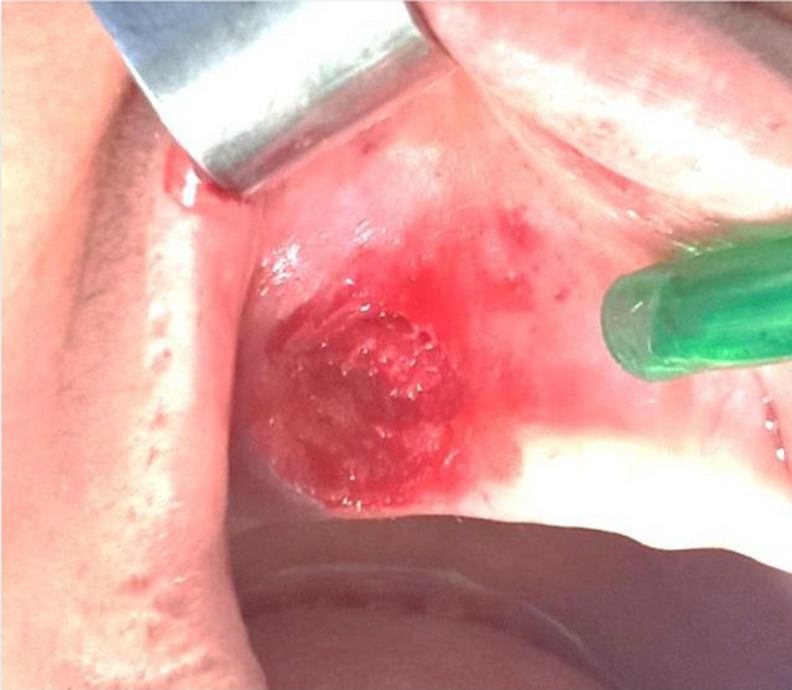
intra-oral view: the site after excisional biopsy and complete curettage

**Figure 3:**
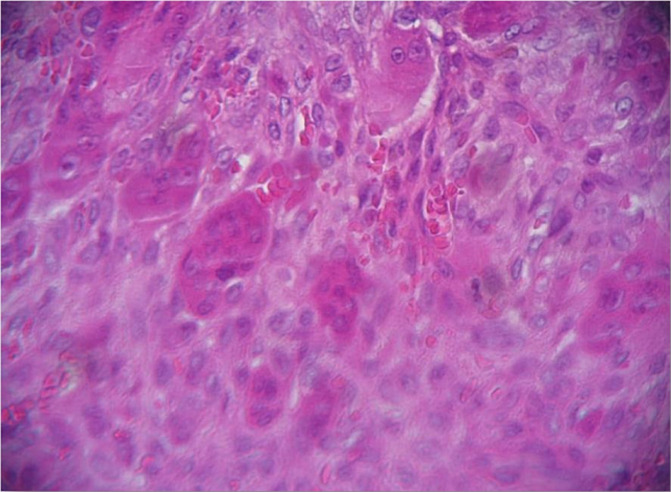
histological study showing the predominance of multinucleated giant cells surrounded by mononuclear stromal cells and extravasated red blood cells

**Therapeutic interventions:** surgical removal was performed under local anesthesia. The lesion was separated from the adjacent tissue and excised in one piece (Figure 2). Primary closure was achieved with controlled hemorrhage. The excised lesion was then sent for histopathological study and showed no spindle cells/inflammatory cells but did reveal the presence of multinucleated giant cells. The microscopic study concluded with a diagnosis of peripheral giant cell granuloma (Figure 3).

**Follow-up and outcome of interventions:** sutures were removed one week later with an uneventful healing process. The patient was then followed up for a period of 2 months which did not show any evidence of recurrence.

**Patient's feedback:** the patient was satisfied with the successful surgery and showed a positive reaction during the follow-up period.

**Patient consent:** a written informed consent was signed by the patient for inclusion and consideration in our current study.

## Discussion

Peripheric giant cell granuloma is an exophytic common lesion occurring in the oral cavity. These perivascular lesions are mainly consecutive to local irritants such as tartar, plaque, incompatible restoration, traumatic tooth extraction, and chronic inflammation [5]. This case report demonstrates a case of traumatic previous extractions that led to the development of PGCG in an adult female patient after extraction of teeth 13 and 14. Although PGCGs could be seen in all age groups, they are most commonly observed in the 40-60 age group. In addition, the PGCGs predilection site is the mandible, rarely occurring in the maxilla [6].

In this current case report, a patient was 60 years old and had a lesion seen in the maxillary canine premolar region. It has been reported that PGCG is about 1.5 times more likely to occur in the mandible than in the maxilla [6]. A study on 62 cases reported that 43 of them occurred in the mandible (69.4%) and 19 (30.6%) in the maxilla [6]. According to Bodner *et al*. the mandible was more affected than the maxilla. In Demirkol *et al*. study, they examined 16 PGCG cases and found that 4 (25%) were seen in the maxilla and 12 (75%) were observed in the mandible [7]. They also reported that only 1 (6.25%) was seen in the maxilla posterior region and 8 (50%) were seen in the mandible posterior region, which explains why the maxillary predilection site of PGCG is rare. This case report describes a PGCG lesion located in the canine-premolar region of the maxilla. The literature has described an occurrence rate of PGCG post-extraction, but the possible traumatic extraction etiology to cause of PGCG is still unclear and not proved. Mighell *et al*. reported a case of PGCG two months after the orthodontic extraction of a deciduous molar. This study suggested that the presence of growth factors in the healing socket may have contributed to the PGCG development and ultimately, its progression into a lesion. Our current clinical case is in concordance with the Mighell *et al*. study where our patient underwent traumatic extractions 1 year before the lesion appeared, which has led to the occurrence of the lesion [8]. Considering the clinical characteristics, fibroma, peripheral ossifying fibroma, hemangioma, epulis, and pyogenic granuloma are all possible diagnoses. When looking at the histological findings that are similar to a brown tumor, aneurysmal bone cyst, and benign osseous dysplasia, it is important to keep them in mind as potential diagnoses [9].

For PGCGs, surgical excision of the mass and preventative measures to eliminate any potential predisposing factors are the recommended treatment. This can include the extraction of any associated teeth in cases of periodontal ligament involvement [9]. Fortunately, recurrence is rare when the entire lesion is completely removed. According to Neville *et al*. recurrence rates ranged between 11-50% in their multiple case series [10]. In our case report, no recurrence was observed following the complete excision of the lesion during a two-month follow-up period. It's important to remember that these granulomas can become quite large if neglected.

## Conclusion

Early detection of peripheral giant cell granuloma on the basis of clinical, radiographic, and histopathological examination enables conservative management with minimal risk to the adjacent hard tissues consisting of complete excision of the lesion in order to minimize further recurrences. It is important that clinicians should be aware that traumatic extractions can be one of the main etiologic factors leading to the occurrence of peripheral giant cell granuloma.
